# Progression of Eales’ disease post-partum and long-term follow-up: a case report

**DOI:** 10.1186/s13256-018-1768-y

**Published:** 2018-10-17

**Authors:** Radu Ivanescu, Ciprian Ivanescu, Kai Januschowski, Augustin Ivanescu

**Affiliations:** 1 Opticlass, Coriolan Brediceanu no. 29, Timisoara, Romania; 2 Eye Clinic Sulzbach, Knappschaft Hospital Saar, An der Klinik 10, 66280 Sulzbach-Saar, Germany; 3 Centre for Ophthalmology, University Eye Hospital Tübingen, Schleichstr. 12, D-72076 Tuebingen, Germany; 4 Knappschaft Eye Clinic, Knappschaft Hospital Saar, 66280 Sulzbach, Germany

**Keywords:** Eales’ disease, Recurrent vitreous hemorrhage, Retinal vasculitis, Retinal neovascularization, Photocoagulation, Dexamethasone implant

## Abstract

**Background:**

Eales’ disease is a difficult to treat idiopathic form of retinal vasculitis. We present such a case with peculiar epidemiology in which the only efficient therapeutic approach in order to maintain functional vision was repeated intravitreal dexamethasone implants (Ozurdex®). To the best of our knowledge this is the first report of this kind in the literature.

**Case presentation:**

This is the case of a 40-year-old white woman presenting in 2008 with recurrent floaters accompanied by blurring of vision in her right eye and severe visual loss in her left eye caused by retinal detachment; her age at presentation was 30 years. A diagnosis of Eales’ disease was made by exclusion. She was stable until 2011 after childbirth (cesarean section). Despite aggressive systemic treatment with steroids, azathioprine, and mycophenolate mofetil, progression of the disease was noted that could only be controlled with repeated intravitreal dexamethasone implants (Ozurdex®) injected in her right eye. Her left eye underwent vitrectomy with silicone oil tamponade for tractional retinal detachment with satisfactory anatomical result but vision was limited to light perception.

**Conclusions:**

In our case the systemic therapy was much less effective than the intravitreal sustained-release dexamethasone implant (Ozurdex®) for reducing recurrences and halting disease progression. We believe that recurrent and severe cases could benefit from this addition to the classic treatment armamentarium.

**Electronic supplementary material:**

The online version of this article (https://doi.org/10.1186/s13256-018-1768-y) contains supplementary material, which is available to authorized users.

## Background

Eales’ disease, first described by the British ophthalmologist Henry Eales in 1880, is characterized by three overlapping stages of venous inflammation (vasculitis), occlusion, and retinal neovascularization [1]. Diagnosis is mostly clinical and requires exclusion of other systemic or ocular conditions that could present with similar retinal features. In recent years, immunological, molecular biological, and biochemical studies have indicated the role of human leukocyte antigen, retinal autoimmunity, *Mycobacterium tuberculosis* genome, and free radical-mediated damage in the etiopathogenesis of this disease [2]. However, the etiology appears to be multifactorial. Regarding disease staging there is no standard classification system to date. Charmis proposed a four-stage grading system in 1965 based on morphological aspects alone, which was not popular due to the overlap of stages found in typical clinical settings. More recently, Saxena and Kumar proposed a new system based on visual outcomes connected to disease severity [3]:

- Stage 1 is periphlebitis of small (1a) and large caliber vessels (1b) with superficial retinal hemorrhages.
- Stage 2a denotes capillary non-perfusion and stage 2b neovascularization elsewhere/of the disc.
- Stage 3a is fibrovascular proliferation and stage 3b is classified as vitreous hemorrhage.
- Stage 4a is tractional/combined rhegmatogenous retinal detachment whereas stage 4b is rubeosis iridis, neovascular glaucoma, complicated cataract, and optic atrophy [3].

The management of Eales’ disease depends on the stage of the disease and consists of medical treatment with orally administered corticosteroids in the active inflammatory stage and laser photocoagulation in the advanced retinal ischemia and neovascularization stages [4]. Eales’ disease is usually diagnosed in healthy young men. Few studies considering intravitreal steroid injections exist; however, they indicate a positive effect [5]. Data about slow-release steroid implants and data on female patients are very rare. In this case report we describe the clinical findings, diagnostic management, and therapeutic management with slow-release steroid implants and the long-term evolution of an otherwise healthy 32-year-old woman who we eventually diagnosed as having Eales’ disease that worsened after childbirth.

## Case presentation

A 32-year-old white woman presented to our clinic in 2009 complaining about floaters in her right eye and severely reduced visual acuity in her left eye. Her visual acuity was 20/20 in her right eye and hand motion perception in her left eye. A fundus examination revealed vitreous hemorrhage in her right eye and retinal detachment in her left eye. She was admitted and emergency surgery was performed.

There was no previous ocular trauma or history of other eye diseases/eye surgery. There was no family history of eye diseases.

### Diagnostic and therapeutic approaches

Scatter laser photocoagulation was performed in her right eye and at a follow-up visit in July 2009 a fluorescein angiography was performed. This showed neovascularization of the optic disc, and non-perfusion in the peripheral retina with peripheral neovascularization and central neovascularization that involved the temporal part of the macula. The best-corrected visual acuity in her right eye was 20/20; intraocular pressure was 12 mmHg (Goldmann applanation tonometry). Perivasculitis with periphlebitis and vitreous hemorrhage was also noted. An optical coherence tomography (Heidelberg Spectralis®) scan showed atrophy of the internal retinal layers corresponding to the area of non-perfusion and a thickening of the internal limiting membrane.

Laser treatment was performed and 1 month later neovascularization of the optic disc decreased but residual perivasculitis was noted. An optical coherence tomography scan was stable except for further thickening of internal limiting membrane and incipient epiretinal membrane formation. Other laboratory workups including C-reactive protein (CRP), erythrocyte sedimentation rate (ESR), angiotensin-converting enzyme (ACE), interleukin-2 (IL-2) receptor, *Treponema pallidum* hemagglutination assay (TPHA), partial thromboplastin time (PTT), Venereal Disease Research Laboratory (VDRL), and interferon-gamma release assay (QuantiFERON-TB Gold) revealed no significant abnormalities. Thorax radiography revealed no suspicious lesions indicating sarcoidosis or tuberculosis. A further review of her systems was unremarkable. There were no accompanying systemic signs and symptoms except lower back pain and marginally reduced hip mobility that was diagnosed as human leukocyte antigen-B27 (HLAB27) positive sacroiliitis.

In 2010 our patient became pregnant and her disease course was stable for the duration of the pregnancy with no additional treatment needed.

At 25 months from baseline (1 month after childbirth) our patient presented with recurrence of vitreous hemorrhage and worsening of visual acuity (20/40). On fluorescein angiography there was severe perivasculitis, optic disc neovascularization, and increased area of non-perfusion. More scatter laser photocoagulation was performed and orally administered prednisone 1 mg/kg with a slow taper was started. However, stability in angiography and clinical findings was not achieved. At 28 months from baseline, she was started on methotrexate 20 mg/week and prednisone 10 mg/day. Because of hepatotoxicity, methotrexate was tapered and at 40 months from baseline azathioprine 100 mg and mycophenolate mofetil 1.5 mg was prescribed. Despite aggressive systemic therapy, the disease course was relentless. At 43 months from baseline, enlargement of non-perfusion area was noted and intense perivasculitis was noted on fluorescein angiography. On an optical coherence tomography scan loss of internal retinal layers was noted that involved the temporal half of the fovea. An epiretinal membrane with slight papillomacular traction was observed. At 44 months from baseline a dexamethasone sustained-release implant (Ozurdex®, Allergan; Bucharest, Romania) was injected. This halted the disease, with a best-corrected visual acuity of 20/20 progression and since then ten steroid implants have been used. We recommended injection every 4 months but because of the financial burden for our patient (no reimbursement for Ozurdex®) we injected based on worsening of angiogram, optical coherence tomography, and/or recurrent vitreous hemorrhage.

She stopped mycophenolate mofetil at 74 months from baseline after a slow taper and azathioprine at 75 months from baseline.

She underwent uneventful cataract surgery at 67 months from baseline for her right eye and at 48 months from baseline for her left eye preceded by vitrectomy for retinal detachment in 2009 and second vitrectomy with silicone oil injection for proliferative vitreoretinopathy at 85 months from baseline. In November 2016, fluorescein angiography was stable with minimal vasculitis, no active neovascularization, and no progression of non-perfused areas for her right eye. The last Ozurdex® was implanted 109 months from baseline. Although there are studies showing intraocular pressure increase after Ozurdex® [6], in our case there was no need for intraocular pressure-lowering medication as throughout the follow-up period pressures were between 10 mmHg and 18 mmHg. Additional files provide comparison between fluorescein angiography before Ozurdex implantation and after the last implant was used (Additional file 1) and optical coherence tomographies showing progression from baseline to the last follow-up visit (Additional file 2). Widefield imaging and composite fundus photography can be observed in Additional file 3. Additional file 4 provides an overview of the therapeutic approaches (case report timeline.

### Outcomes

Follow-up examinations were aimed at picking up early signs of disease progression and we gave timely injections of Ozurdex® in order to preserve a functional retina taking into account the financial burden for our patient. In our case the systemic therapy was much less effective than the intravitreal sustained-release dexamethasone implant for reducing recurrences and halting disease progression. Currently our patient is completely functional at work with 20/20 visual acuity for her right eye and light perception for her left eye. Despite the reduced visual field, according to her statements, she is able to perform all daily activities with ease.

## Discussion

Our case is peculiar due to the higher prevalence of this disease in young men and lack of previous exposure to tuberculosis that is associated with Eales’ disease. To the best of our knowledge this is the first case describing continued use of a dexamethasone intravitreal slow-release system in a female patient. Also there was a severe disease course and limited response to aggressive systemic treatment that worsened after childbirth with recurrent vitreous hemorrhages and progressive loss of functional retina. Disease control could only be guaranteed by systemic steroids (7.5 mg/day) and intravitreal sustained-release dexamethasone implant (Ozurdex®). The clinical picture for this patient fits the findings associated with Eales’ disease that it is a diagnosis of exclusion. All laboratory workups were negative for any autoimmune or hematological diseases that could cause vitreous hemorrhage or posterior uveitis. Disease onset typically occurs during the second to third decade of life and this is also true for our patient [7–9]. The pathogenesis of vaso-occlusion is still not fully understood. However, it is generally accepted that the inflammatory process leads to retinal ischemia that causes neovascularization. The newly formed vessels bleed easily, and this plays a major role in decreasing the patient’s vision [10]. Of patients with Eales’ disease, 80% develop neovascularization either on the disc or elsewhere, most commonly in the peripheral retina [10]. A bilateral involvement of the eyes is common [7–9].

Saxena and Kumar have suggested a staging system useful in classifying and assessing the severity of the disease [3]. According to this classification our patient can be labeled as stage 3b in her right eye and stage 4b in her left eye.

Successful treatment of Eales’ disease is achieved by controlling retinal perivasculitis and decreasing the risks of vitreous hemorrhage from new blood vessels [11]. For almost all neovascularizations in the retina, regardless of the cause and if there is no contraindication, laser photocoagulation, which prevents proliferation of new vessels in the ischemic retina, is the mainstay of treatment [12]. Observation and corticosteroids are the ideal treatment options for inactive retinal vasculitis and active perivasculitis, respectively [1]. In our case the combined approach of laser photocoagulation, systemic steroids, and intraocular dexamethasone implant slowed and then halted the disease course maintaining useful vision in her right eye. However, as we are observing increased formation of epiretinal membrane our patient may benefit from vitrectomy and membrane peeling in the future. Ozurdex® has proven to be safe in some studies in vitrectomized eyes for the treatment of diabetic macular edema [13] but one has to expect, in our opinion, that more injections would be needed, which would impact the patient’s financial involvement (Ozurdex® is not reimbursed in Romania). In the future, even longer acting sustained-release corticosteroid implants could be an option for our patient.

## Conclusions

We believe that cases of recurrent and severe Eales’ disease that respond poorly to laser photocoagulation and systemic treatment could benefit from the addition of intravitreal sustained-release dexamethasone implant (Ozurdex®) to the classic treatment armamentarium.

## Additional files

Additional file 1: **Figures 1 and 2**. Fluorescein angiographies comparing before OzurDex (Additional file 2: Figure 3) and last angiography after 10 OzurDex implants were used in a period of 5 years. (JPEG 809 kb)

Additional file 2: **Figure 3**. Foveal OCT follow-ups showing baseline image (top), before first OzurDex (middle) and after 10 Ozurdex implants were used (bottom). (JPEG 1.10 mb)

Additional file 3: **Figure 4**. Widefield imaging (Optos Daytona) of right eye. **Figure 5**. Composite fundus photography showing left eye (top) and right eye (bottom). (JPEG 1.30 mb)

Additional file 4: **Figure 6**. Case Timeline. (JPEG 456 kb)
