# Supplementary figures and images for: Progression of Eales’ disease post-partum and long-term follow-up: a case report
Source: J Med Case Rep. 2018 Oct 17;12:310. doi: 10.1186/s13256-018-1768-y (PMC6192282; doi:10.1186/s13256-018-1768-y)

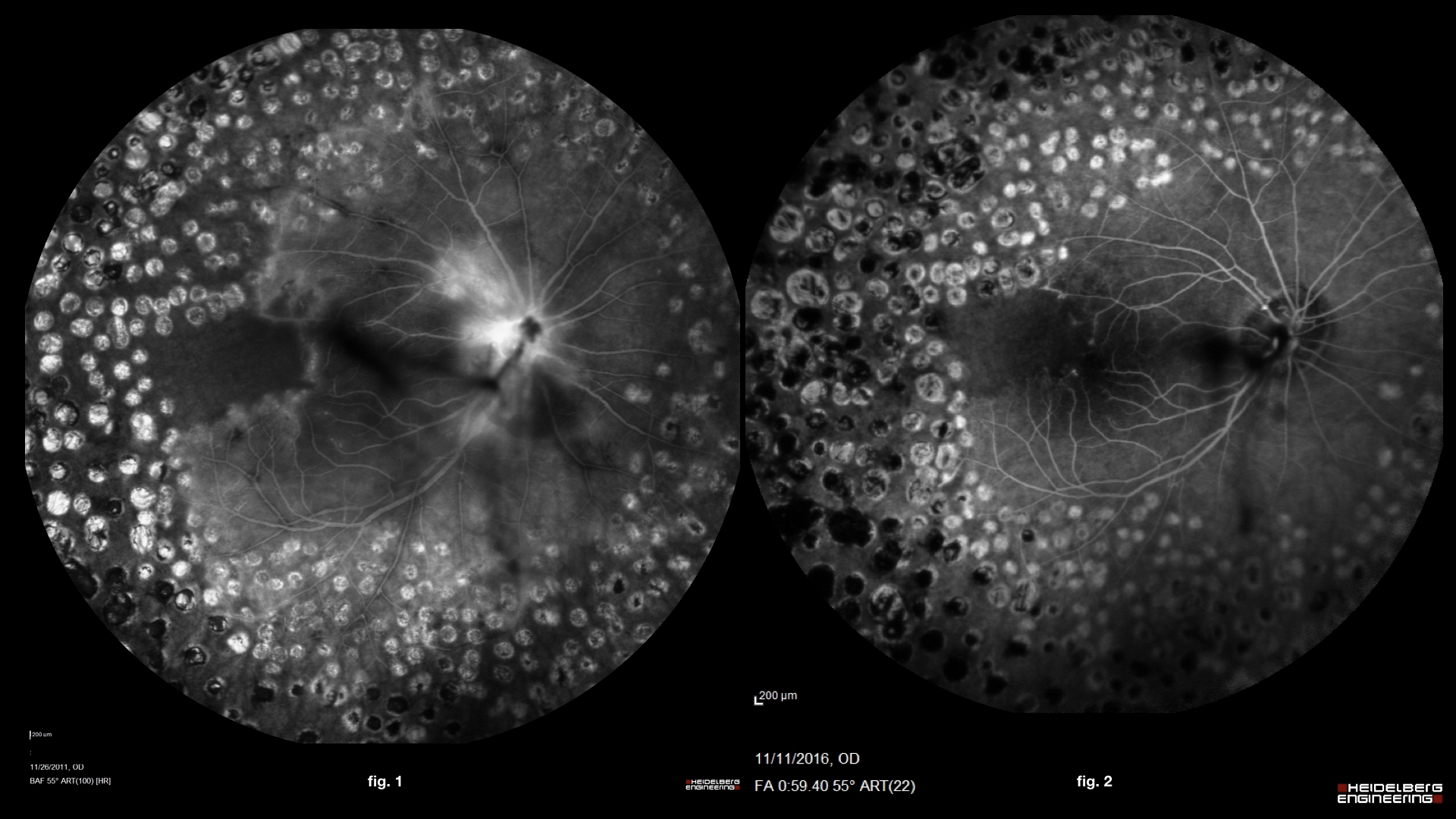

Supplement: Supplementary file 1 — Figures 1 and 2. Fluorescein angiographies comparing before OzurDex (Additional file 2: Figure 3) and last angiography after 10 OzurDex implants were used in a period of 5 years. (JPEG 809 kb) [file 13256_2018_1768_MOESM1_ESM.jpeg]

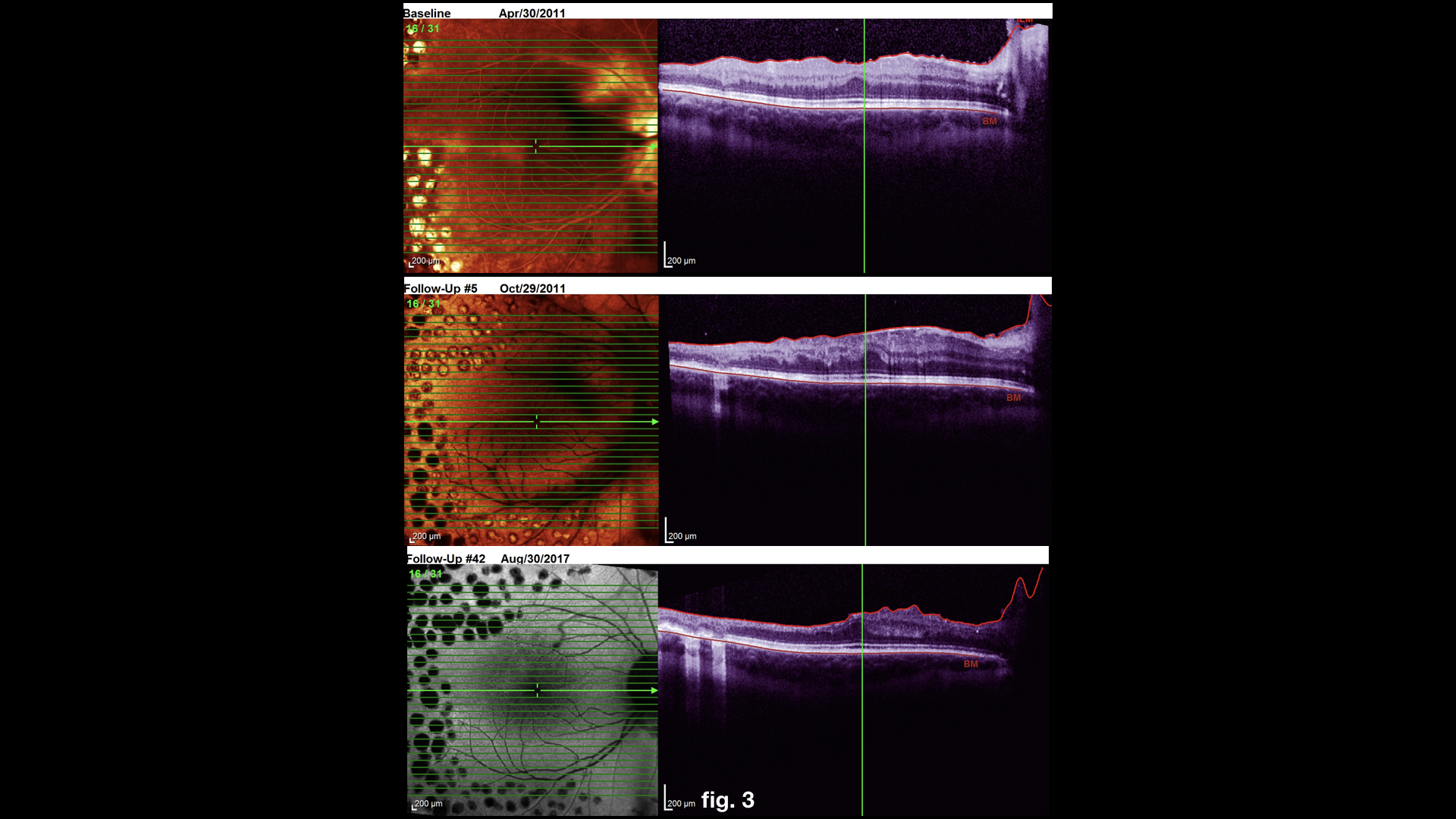

Supplement: Supplementary file 2 — Figure 3. Foveal OCT follow-ups showing baseline image (top), before first OzurDex (middle) and after 10 Ozurdex implants were used (bottom). (JPEG 1.10 mb) [file 13256_2018_1768_MOESM2_ESM.jpeg]

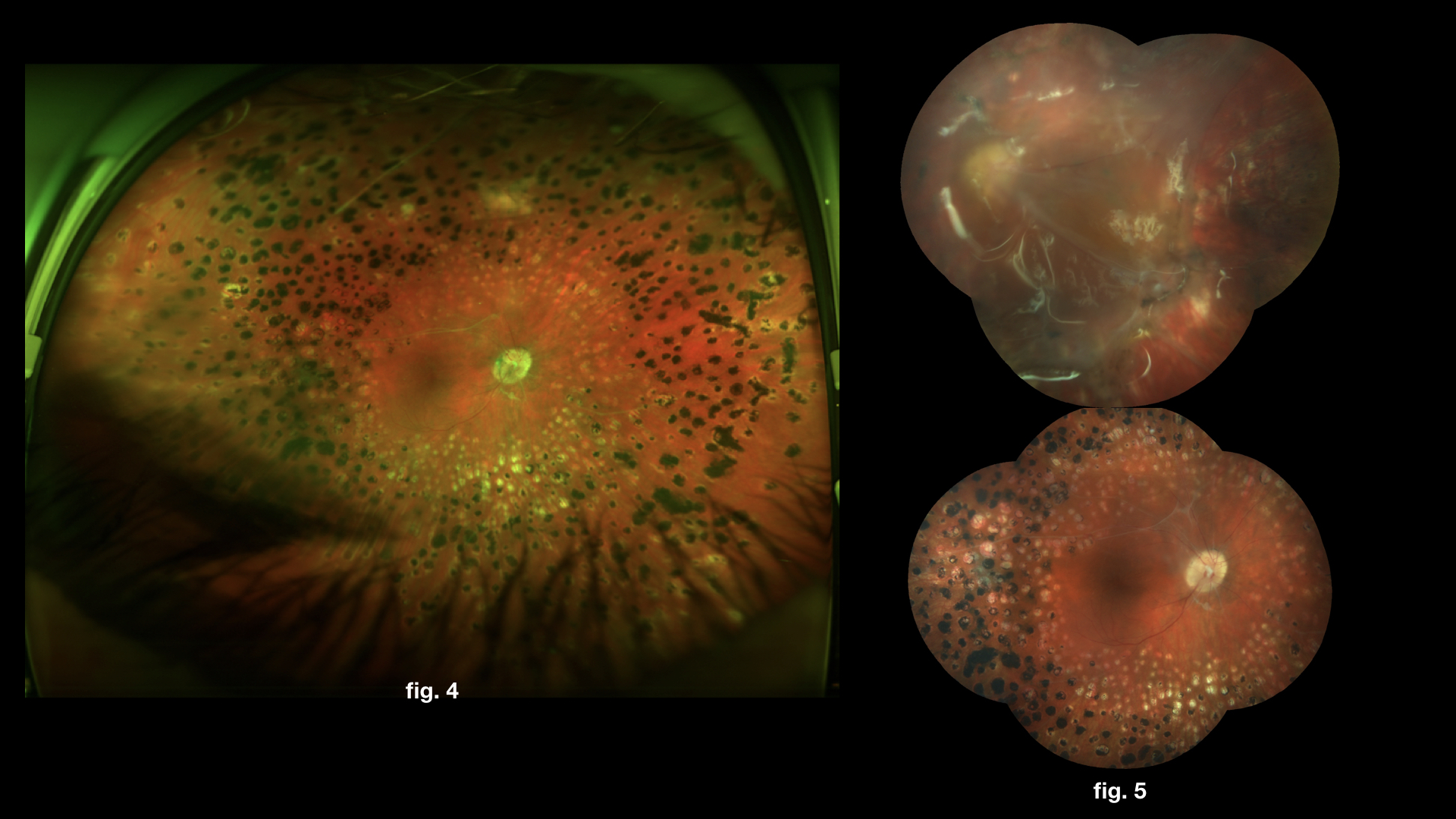

Supplement: Supplementary file 3 — Figure 4. Widefield imaging (Optos Daytona) of right eye. Figure 5. Composite fundus photography showing left eye (top) and right eye (bottom). (JPEG 1.30 mb) [file 13256_2018_1768_MOESM3_ESM.jpeg]

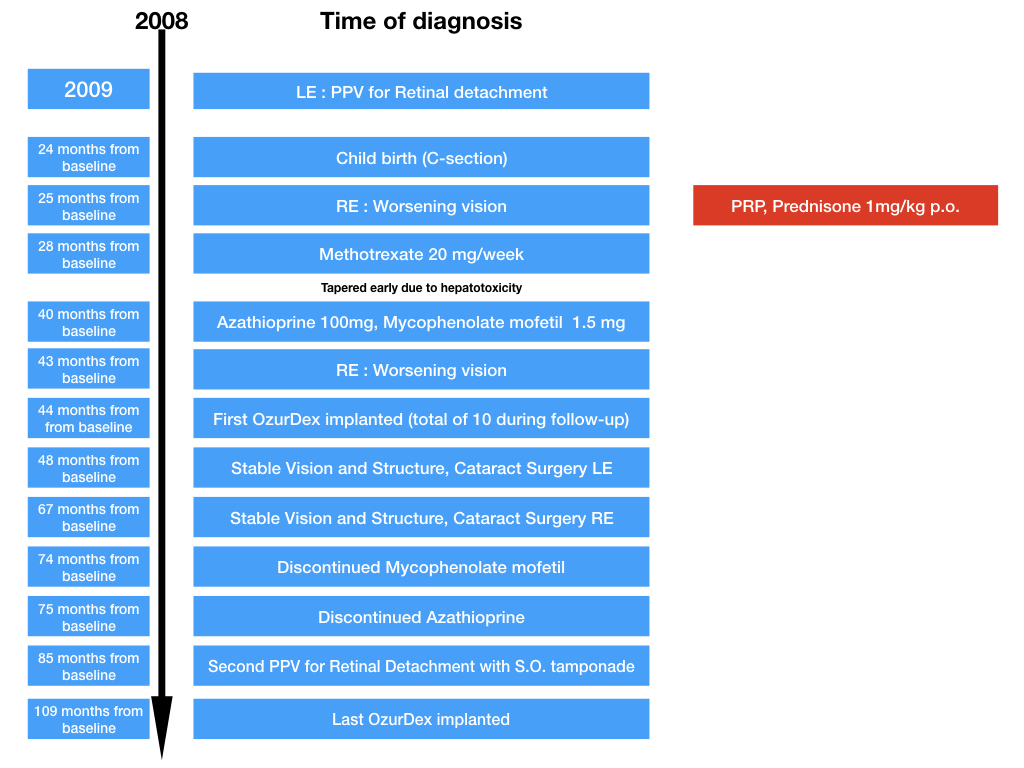

Supplement: Supplementary file 4 — Figure 6. Case Timeline. (JPEG 456 kb) [file 13256_2018_1768_MOESM4_ESM.jpeg]
